# ATLAS: Mapping ATtention’s Location And Size to probe five modes of serial and parallel search

**DOI:** 10.3758/s13414-024-02921-7

**Published:** 2024-07-09

**Authors:** Gregory Davis

**Affiliations:** Department of Psychology, University of Cambridge, Downing Street, Cambridge, CB2 3EB UK

**Keywords:** Attention, Visual Search, Parallel, Serial, Spotlight, ATLAS

## Abstract

Conventional visual search tasks do not address attention directly and their core manipulation of ‘set size’ – the number of displayed items – introduces stimulus confounds that hinder interpretation. However, alternative approaches have not been widely adopted, perhaps reflecting their complexity, assumptions, or indirect attention-sampling. Here, a new procedure, the ATtention Location And Size (‘ATLAS’) task used probe displays to track attention’s location, breadth, and guidance during search. Though most probe displays comprised six items, participants reported only the single item they judged themselves to have perceived most clearly – indexing the attention ‘peak’. By sampling peaks across variable ‘choice sets’, the size and position of the attention window during search was profiled. These indices appeared to distinguish narrow- from broad attention, signalled attention to pairs of items where it arose and tracked evolving attention-guidance over time. ATLAS is designed to discriminate five key search modes: serial-unguided, sequential-guided, unguided attention to ‘clumps’ with local guidance, and broad parallel-attention with or without guidance. This initial investigation used only an example set of highly regular stimuli, but its broader potential should be investigated.

## Introduction

Visual search tasks provide some of experimental psychology’s most powerful and widely used performance measures (Wolfe & Horowitz, 2017; Wolfe, 2020). Typically, these experiments measure response times (RTs) to detect single targets among nontarget items; the key performance measures, search slopes, measure increases in RT that occur as the number of items presented, the set size, is increased. In classic studies of attention, search-slopes were used to distinguish between fundamentally different modes of searching: serial, attention-based search and parallel, ‘preattentive’ processing (e.g., Shiffrin, 1977; Treisman & Gelade 1980). However, while still informing models of attention (Wolfe & Horowitz, 2017), slopes now primarily serve as general metrics of processing efficiency. These are useful for comparing detectability of stimuli (e.g., direct vs. averted eye gaze; Ramamoorthy et al., 2019), effects of stimulus heterogeneity (Buetti et al., 2016) and other factors (e.g., Wolfe & Horowitz, 2017). Nonetheless, attention selection and shifting remain core components of human search. To understand search processes better, we may therefore need to revisit the serial-parallel distinction (Liesefeld & Müller, 2020). Here, noting limitations of standard visual-search measures for studying attention, an alternative approach is introduced, designed to track the size, location and guidance of visual attention during search.

The shift away from addressing parallel versus serial processes in search has been motivated, in part, by an inability of search-slope analyses to distinguish parallel from serial processing. While shallow slopes within a narrow range (< 10 ms per item) are still broadly agreed to reflect highly efficient parallel processing (e.g., Wolfe, 1998), there is little agreement as to how steeper slopes should best be modelled (e.g., Moore & Wolfe, 2001; Townsend et al., 2011; Wolfe, 1998). They may either reflect inefficient parallel processing (evidence accumulation and guidance of attention toward the target) or serial, unguided attention to ‘clumps’ of (one or more) items, or both (Liesefeld & Müller, 2020; Palmer, 1995; Wolfe, 2020; Wolfe & Horowitz, 2017). As a result of the set-size and search-slope paradigm’s limitations, many visual search experiments conducted each year are therefore left in conceptual ‘limbo’ regarding whether inefficient search is governed by noisy-parallel or serial processes (e.g., Palmer, 1995; Kristjánsson, 2015). These processes are further distanced from the capabilities of current methods by hybrid parallel-serial mechanisms in Wolfe’s Guided Search models (‘car wash’ processes with serially triggered but parallel-processed evidence accumulators; Wolfe, 2006). Though ingenious analyses of RT distributions have continued to advance understanding of search mechanisms (e.g., Buetti et al., 2016; Moran et al., 2016; Wolfe et al., 2010), the standard set size and search slope paradigm still suffers from key limitations that preclude effective discrimination of serial and parallel processes, or the role of attention.

### Set size: Limitations and alternatives

Search slope analyses compare performance across set sizes and assume that search at larger set sizes recruits the same processes as at smaller set sizes. Notionally, by varying set size, typical visual search experiments manipulate the number of items that the participant must process to find the target. However, comparisons across set sizes are plagued by stimulus confounds (see, e.g., Thornton & Gilden, 2007). To illustrate, consider set size 1 (displays presenting a single item) versus set size 6 (displays of six items, as employed here). Those two conditions differ not only in terms of the factor of interest (how many items the participant may process simultaneously) but also in terms of the perceptual noise generated by the onsets of multiple items (e.g., Eckstein et al., 2000), lateral interactions (Veríssimo, et al., 2021) and the forced spreading of spatial attention across the display. These nuisance variables may overwhelm attention guidance by all but the most efficient guidance processes, making flat search slopes a particularly conservative estimate of what may be processed in parallel.

Second, set-size manipulations also address attention dynamics and guidance mechanisms only very indirectly. Serial and parallel process models with fundamentally different architectures can yield identical patterns of expected mean response times across set sizes even for highly inefficient search (Townsend & Nozawa, 1995; Townsend et al., 2011). Set size manipulations, instead, may be better tailored to address practical questions about ‘effective’ capacities (e.g., of the form ‘how well can participants process N simultaneously presented items for characteristic X’). Though they remain a key component of cognitive psychology’s toolkit, it may be time to develop complementary measures to underpin the sophisticated modelling often applied to these questions (e.g., Moran et al., 2016).

While set size cannot diagnose it, the importance of distinguishing parallel from serial contributions to search is retained in influential models (e.g., Wolfe’s Guided Search models, 1998, 2020), reinforcing its importance (e.g., Townsend, 1990; Townsend & Wenger, 2004). To help address this mismatch between set size manipulations and theory aspirations, a range of behavioural measures have been proposed to distinguish serial from parallel search, including 2:1 search-slope ratios (Treisman & Gelade, 1980), target-position effects (e.g., Bricolo et al., 2002) and computational models based on RT distributions (e.g., Moran et al., 2016; Narbutas et al., 2017; Sung, 2008). Among the most ambitious and rigorous approaches has been developed by Townsend and colleagues (Systems Factorial Technology; in particular, Double Factorial Design; Houpt & Townsend, 2012; Lowe et al., 2019; Townsend & Nozawa, 1995; Townsend & Wenger, 2004). They attempted to establish a complete mental chronometry of visual search, though those comparisons required two targets, an assumption of selective influences on sub-processes (Eidels et al., 2011) and commitments to considering pre-specified types of models (Harding et al., 2017). The ingenuity and thoroughness of each of these approaches is beyond dispute, but they have not been widely adopted. Instead, much visual search work has continued to manipulate set size.

Another recently developed method for distinguishing serial, self-terminating search from some types of parallel search, has examined dual-target compatibility effects (faster RTs for compatible than incompatible targets – Lee et al., 2021). That approach seems to be based (though not stated explicitly) on redundant-target studies that kept set size constant (circumventing some stimulus-based confounds of set size manipulations) and varied the number of targets per display (e.g., Grice & Canham, 1990; Mordkoff & Yantis, 1991). Redundant target effects were not suited to distinguishing serial from parallel search: Lee et al.’s procedure may yet do so, while minimising commitments to particular models and requiring only minor alterations to standard search tasks. That approach was not adopted here as it requires search for two targets, immediate self-termination in serial search for the measure to distinguish serial from parallel processes. Further, it remains unclear whether dual-target compatibility effects would be expected for all parallel models: load or ‘dilution’ effects may minimise flanker interference (Chen & Cave, 2013; Tsal & Benoni, 2010, Yeshurun, 2019).

### Distinguishing five modes of search

**Fig. 1:**
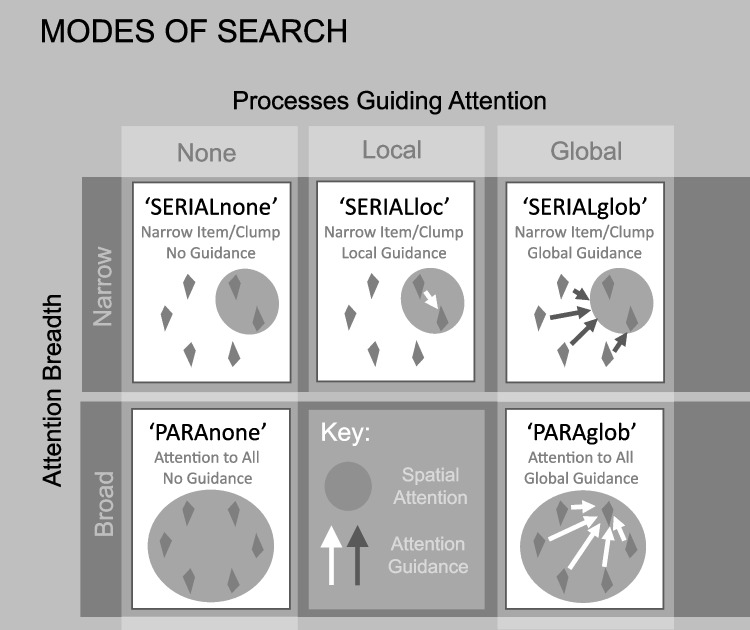
Five modes of search, distinguished by Attention Breadth (rows) and Attention Guidance (columns). Top row, SERIAL narrow attention to single items or small ‘clumps’, left to right: ‘SERIALnone’, no guidance, ‘SERIALloc’, local guidance by processing of attended items, ‘SERIALglob’ guidance via global, parallel processing. Bottom: PARA: broad, parallel attention to all items, ‘PARAnone’, no guidance, ‘PARAglob’ guidance via global, parallel processing

The above measures are generally based on distinguishing predictions of parallel diffusor processes versus of unguided serial selection of one item at a time, with only the latter explicitly involving attention. Indeed, the defining difference between the two polar-opposite versions of these process (Townsend et al., 2011) is conventionally construed as the breadth of parallel guidance of attention (broad vs. none) without explicit reference to attention itself. However, as Liesefeld and Muller (2020) noted, human attention need not respect serial-parallel dichotomies that ignore characteristics of covert attention. The spatial-extent of attention – the size of what we may loosely refer to as the attention ‘window’ – may vary continuously across and within search tasks. Accordingly, measuring spatial attention-guidance contributions alone will not distinguish between five distinct modes of parallel search guidance considered here (see Fig. 1); breadth of spatial attention is also key.

Figure 1 illustrates the five candidate modes of search considered here. Modes differ, in that figure, along two dimensions. The spatial extent of processing involved in attention *guidance* varies across columns: left column: no attention guidance toward the search target, centre column: attention guidance by local parallel processing only, right column: global guidance by parallel processing of all items. Modes of search also differ, across rows, in terms of the breadth *of spatial attention* (N.B.: of attention, not of item processing in general). The top row illustrates search modes characterised by *narrow, sequentially applied* attention, and the bottom row, broad, *simultaneously applied* attention. Note, for simplicity, these five modes do not distinguish attention to one item at a time from attention to a small clump of items at a time (though the measures described here still do so); both are considered examples of ‘serial’ attention (broadly construed) in the strict sense of sequential selection and processing of items by narrowly focussed attention (each completed in some sense, prior to processing of the next item/clump). In the labels for each mode, below, the word in uppercase standard font denotes whether attention is narrow and sequential (‘SERIAL’) or broad and simultaneous, attending items in parallel (‘PARA’), while the word in lowercase, denotes the spatial extent of processing that guides attention: none (‘none’), local only (‘loc’), or global processing of all items (‘glob’).

The top-left of Fig. 1 illustrates a mode of search, ‘SERIALnone’, in which attention is narrowly focussed (on one item or a small ‘clump’ of items, at a time) and selects each item/clump *sequentially* and with *no guidance* toward the target by parallel processing. Lacking guidance, attention is no more likely to choose an item/clump that contains the target than one that does not (though preferentially drawing from previously unsearched items). In this mode, if an attended clump comprises the target, attention is not guided toward it. Rather, the clump is attended until a threshold of perceptual evidence for or against the presence of a target among the attended items, is reached.

The upper, middle panel similarly illustrates a mode of search (‘SERIALloc’) via sequential application of narrow attention, but this time *with local guidance*. As for the first mode, attention again selects a clump of items ‘randomly’ (in the sense of ‘without parallel-processing based guidance toward the target’) but then *local* competition between items in the attended clump guides attention toward the target if it is a member of the currently attended clump. The right, upper panel illustrates narrow, sequential attention *with global guidance* (SERIALglob). In this mode, global parallel processing outside the spatial scope of attention operates (independently of attention) to guide narrow attention spotlight toward the target (or at least to increase the probability that attention selects the target). SERIALglob is akin to examples of guided search. Note that, as the approach adopted here indexes attention breadth and location, not search RTs, it treats Guided Search model’s hybrid ‘car wash’ (accumulators with serially triggered, asynchronous onsets, but parallel processing) as serial attention (Wolfe, 2006).

Beyond these three modes of search with narrow attention, there are also, of course, two corresponding modes in which attention is broadly distributed to most, or all search display items. On the bottom left of Fig. 1, is illustrated ‘PARAnone’ – a mode of search characterised by *broad, simultaneous* attention with *no guidance* of attention assumed (i.e., attention-based parallel perception). The notion here is that the display is broadly attended and processed in parallel until sufficient perceptual information has been accrued to make a present/absent decision about the target in a search display. Finally, the bottom right panel of Fig. 1 illustrates a mode of search characterised again by *broad, simultaneous attention* to most/all display items but now *with* guidance of attention by global, parallel processing: ‘PARAglob’. Presumably, following guidance in this mode, attention’s broad spatial distribution may collapse to the size and position of the item identified as a likely target.

Further, human observers’ search is not necessarily restricted to any of the five broad modes of search described here. It may blend combinations of the above or use one mode in an initial phase of searching each display, then switch to another. However, as the more modest aim of the current work was to develop tools to characterise search in terms of its predominant or most applicable mode (of the five considered) at various stages of processing across trials, these possibilities are accepted as limitations.

### Limitations when characterising the attention window: Spotlights and gradients

Another limitation of the current work that should be discussed is that it relies on crude characterisation of the spatial extent of attention during search. There remains little agreement as to the qualitative shape(s) of the spatial attention’s distribution(s) across space (Yeshurun, 2019). Some classic models drew parallels between the attention window and spotlights (fixed or variable: Eriksen & St. James, 1986; Posner, 1980), suggesting fairly even distribution of attention resource within a circumscribed region of visual space. Others suggested Gaussian gradients (LaBerge & Brown, 1989), on which attention allocation reduces with increasing distance from the location of peak attention. On these latter models, there is no hard edge to attention’s spatial extent and no non-arbitrary way to define the size of the attention ‘window’. Indeed, the term ‘window’ does not fit these latter models well.

The difficulty of characterising attention’s breadth is further complicated in that these simple models do not incorporate effects of object- and figure-ground segmentation (e.g., Duncan, 1984), or specify how measured spatial distributions might differ by assay method (e.g., Yeshurun, 2019) and perceptual load (see, e.g., the attentional ‘functional visual field’ – Hulleman & Olivers, 2017; Wolfe, 2020). Given such uncertainty over attention’s spatial distribution, the goal of the methods introduced here was to support basic distinctions of breadth (between narrow and broad attention) but not to determine spatial attention’s precise shape. The term ‘window’ is therefore employed here to refer to attention distribution without commitments to attention’s precise spatial distribution shape.

Given this uncertainty, one might conclude that, in boundary cases, there would be no sharp distinction between PARAglob and SERIALglob (both parallel guidance, differing in terms of attention breadth). To illustrate, suppose that attention was largely devoted to one item, but a tiny amount of the remaining attention resource was spread evenly and very thinly across the remaining items. No measure could distinguish *virtually zero* attention to items from *zero* attention to items (as discussed earlier), so it must be conceded that the approach adopted here will fail to distinguish them. Accordingly, the five modes discussed here must be considered broad, pragmatically assigned categories without (necessarily) perfectly sharp boundaries. That need not dissuade us, however, of the key importance of distinguishing broad, fairly even attention from narrow attention, with little or no attention paid to the rest of the display at any given moment.

### ATLAS: Sampling attention’s distribution using restricted peak choices

The current work trialled a new approach for profiling attention’s distribution and dynamics within multi-item displays. This approach, the ATtention Location And Size (ATLAS) task, uses participants’ free choices to estimate the location and spatial extent of attention when processing multi-item displays. It is described in outline first, then in more detail with respect to its specific role here: revealing the distribution and guidance of attention during search.

**Fig. 2:**
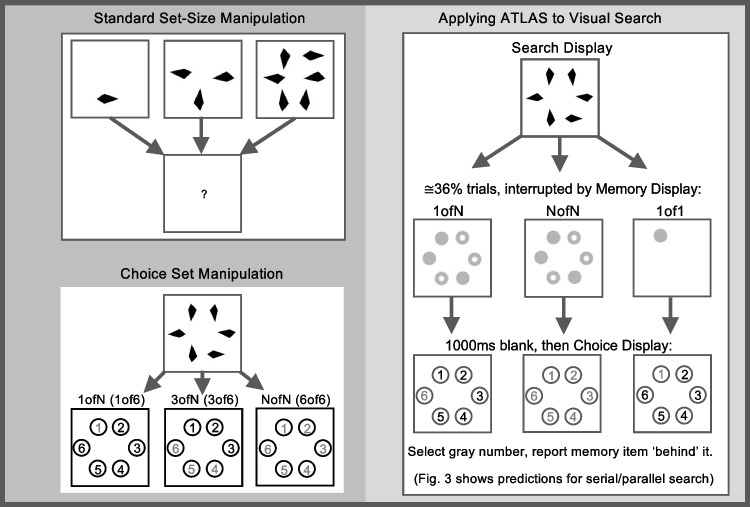
**Left:** basic features of conventional set-size manipulation (top) and ATLAS (bottom). In set size manipulations, physical differences between small and large set sizes constitute substantial confounds that undermine the paradigm. In ATLAS, Search Displays are identical across conditions but are interrupted by Choice Displays. Observers then freely choose the number of the item they best remembered but can only choose green numbers (here, illustrated in gray). **Right panel:** Extension of ATLAS to measure spatial attention during visual search. Observers search displays of six items, sometimes interrupted by a memory display to probe attention in search display. Stimulus variables were closely matched across conditions, in all but one condition (designed, in part, to probe effects of such variation)

When processing any multi-item display, the precision and reliability with which different items are processed will likely vary. Conventional set size approaches (to search, visual working memory, scene perception, etc.) typically assay this distribution by varying the total number of items presented at once in a task display (‘set size’: Fig. 2, top-left). This brings associated limitations, described earlier. In contrast, ATLAS uses a ‘restricted peak choice’ approach (Fig. 2, bottom-left), which presents the same search displays across conditions and probes the distribution of attention within those displays. To do so, the items are presented, then replaced by ‘covers’, numbered 1–6. The participant then *freely selects the one display item they ‘noticed most clearly’*. This choice and associated accuracy of report are assumed to sample ‘peak’ attention to a display: its (loosely defined) ‘intensity’ (some reflection of the amount of resource allocated) and its *location* within the display. To sample the attention’s spatial *distribution* more broadly, the degree of choice is also varied across conditions. In each trial, participants may only choose to report an item from among the *green numbered covers*. These numbers constitute the ‘Choice Set’, the size and distribution of which is varied to reveal underlying distributions of attention when processing the display (Fig. 2, bottom-left, illustrates Choice Sets with one, three and six items). The features that, in combination, distinguish ATLAS are, first, participants freely select the one item they judge they are maximally able to report (‘Peak Choice’) and, second, systematic restriction of the number and spatial arrangement of items from which they can choose (the ‘Choice Set’).

### ATLAS: Relationship to other attention probe studies

Previous work has used attention probe designs to study the allocation of attention during visual search. For example, Kim and Cave (1995) used RT and accuracy of responses to attention probes presented during search to index, across a range of short stimulus onset asynchronies (SOAs), the amount of attention resource directed to the target versus to *nontarget* items of the same colour as the target, or of a different colour. That is, attention probes were used to map evolving attention bias toward the target (as the current paper will address later, with respect to Experiments 5a and 5b). This approach can also map subtler properties, such as suppression of attention to regions directly adjacent to the target (Cave & Zimmerman, 1997; Cutzu & Tsotsos, 2003) and have also been extended for use with M/EEG (Luck et al., 1993; Hopf et al., 2006, 2010). Related work has used this approach to examine the tendency of attention to be drawn to salient distractors within a search display (see, e.g., Gaspelin & Luck, 2018) and other complex phenomena in search (e.g., Zhang & Carlisle, 2023, for a recent example). Dot probe experiments (and other similar tasks) therefore have proven strengths with regard to tracking attention bias toward the target or toward a salient distractor. Though they use a dual-task design (as does ATLAS), they effectively minimise task complexity by requiring only a response to a single probe item. This suggests, that provided dual-task challenges for the participant are minimised in this way, these tasks should provide reliable measures of spatial attention.

ATLAS’s ‘1of1’ Condition, described later, is essentially a probe task of this kind. It can in principle offer a measure of attention bias toward the target across trials. Note, however, that such tasks do not, in isolation, estimate attention *breadth* or its influences on measured attention bias (and were not used to do so). Accordingly, while those probe tasks are the model for the 1of1 Condition here, ATLAS includes the 1of1 Condition as part of a broader range of conditions designed to assess both target bias and breadth of attention, to help distinguish the broad modes of search considered here. The use (and comparison) of multiple measures can also help to control for the presence of dual-task conditions, common to all those conditions (as discussed later).

Another approach to attention-measurement during search presents a probe display item at the location of every search display item (on trials when the probe display is presented). These tasks often use variations of ‘whole report’, asking participants to report the features (e.g., colours, orientations) of all display items (e.g., Linke et al., 2011; Watson & Humphreys, 2000; Zhang et al., 2020). This alternative approach has the potential to sample information about the participant’s coding of multiple display items within a single display. Indeed, some such tasks permit participants to choose freely the order in which they report the items (e.g., Zenon et al., 2008; Gaspelin et al., 2015).

This approach, however, is also not used for (or intended to be used for) assaying *breadth* of attention. Intuitively, on such a measure, broader attention would be associated with accurately reporting more items, narrow attention with reporting fewer items. However, reporting more items would necessarily place much larger task-related processing burdens on the participant for broad attention than narrow attention, introducing a confound. This limitation would be particularly exacerbated as the participant’s stated task is to report ‘as many items as possible’. While reporting some items, the participant must still maintain multiple items’ noisy representations in memory and may choose to report items with weaker representations *first* to maximise their report of items overall; this could essentially invert the assumption in that task that tendency to report a particular item reflects *more* initial spatial attention to it. ATLAS adopts a different approach that mitigates these limitations by asking the participant only to report one item, relying instead on variations of the set of reportable items to index the position and spatial distribution of attention.

In summary, ATLAS shares features in common with both of these previous approaches to probing attention in visual search. However, its use of Choice Sets helps it retain part of the key strength of dot/single probe experiments (minimising memory load) and also, potentially, to provide an improved measure (relative to full report paradigms) of attention breadth. Together, these features of ATLAS, as illustrated in the remaining sections of this paper, should help to distinguish the different modes of attention articulated earlier. This capability is demonstrated here only with respect to one example set of search stimuli, but the results motivate further investigation with other stimuli and tasks.

To anticipate the structure of the discussion here, the testing of ATLAS is described in two separate sections. Five key modes of search are distinguished by Breadth of Attention (by row, in Fig. 1) and Breadth of Processing Driving Attention Guidance (in columns). Correspondingly, the discussion here first focusses on measuring attention breadth (Experiment 1a–4b), then attention guidance (Experiments 5a and 5b).

## Using ATLAS to measure attention breadth in search

ATLAS was adapted here to profile spatial attention and its guidance *during visual search*. Note, the key variable of interest was not accrued evidence regarding the search items at any moment, but rather how much attention was being paid to different items at that moment. Therefore, an extension was introduced to the basic paradigm outlined above. While participants searched through Search Displays of a fixed set size (six items), the display was sometimes interrupted by a brief Memory Display (of completely different items) to probe spatial attention during search (Fig. 2, right). The Memory Display was then replaced by a Choice Display and participants chose freely which single Memory Display item they could most easily report, by choosing the corresponding number from the Choice Display. However, their choice was restricted to items in the ‘Choice Set’, for which the corresponding numbers in the Choice Display were green (in Fig. 2, either 1, 3 and 6 numbers).

The Memory task used here was the ‘Rings Task’. In each Memory Display, three rings and three circles were briefly presented (50 ms, see right panel of Fig. 2 for examples, Fig. 5A for complete sequence) or four of one shape, two of the other. Following a blank display (1 s), the Choice Display then appeared, prompting the participant to select the single Memory Display item they noticed most clearly from among those corresponding to green numbered positions. The participant then indicated whether the item they selected was a ring or a circle. ATLAS, like other attention probe studies, is a ‘dual task’ procedure, in which resource allocation to each task may impact performance of the other. Conceding this, ATLAS minimises cognitive loads imposed by the Memory Task and compares performance across conditions (that share dual-task limitations) to mitigate these complications (as discussed earlier).

### Characterising breadth of attention: Three primary conditions: 1ofN, NofN, 1of1

Three conditions were used to ascertain whether attention was broad or narrow (illustrated in Fig. 2, right panel). The first of these conditions presented six Memory Display items and offered no choice: only one Choice Display number was green (pseudorandomly selected) and the participant reported the Memory Display item at that location. That condition, ‘1ofN’ (1 reportable item of N items presented; here, N=6) was equivalent to many standard attention probes; *this constituted an effective performance baseline*, an estimate of average attention to Search-Display items across the display.

In the second condition, NofN, *all* numbers in the Choice Display were green, signalling that the participant could choose to report *any* Memory Display item they perceived most clearly and could most reliably report. Accuracy in the NofN condition estimated *peak* attention in the search display – its ‘intensity’ (amount of allocated attention resource), and location (in each trial when sampled). The attention peak likely shifts constantly during search and cannot be measured with standard behavioural (or perhaps, physiological) probes. However, by allowing participants to choose the item they report, ATLAS leveraged participants’ conscious access to which Memory Display item(s) they noticed most clearly (and, by extension, other factors equated, those they attended most). In extremis, even if attention was focused only on one item when the Memory Display appeared, the participant could have chosen that item’s number and performed near ceiling. Equally, if attending broadly, they may again have chosen the item they best remembered.

Finally, a third Condition, ‘1of1’, displayed only one Memory-Display item per trial, at one of the six locations unpredictably (see Fig. 2, right). The logic of this condition was as follows. If attention was broad (even if unevenly distributed) when the Memory Display was presented, attention should have been strongly exogenously cued to the single Memory Display item. Hence, for broad attention, the 1of1 Condition should, like the NofN Condition, guarantee attention to a reportable Choice Set memory item and performance should be approximately equivalent. Such equivalence depends, of course, upon exogenous cueing properties of the Search and Memory stimuli employed, so must be estimated for each stimulus set (as it was here). However, it should be robust to top-down template settings (see Folk & Remington, 2015; unpredictable onsets). This expected outcome differed from that for narrow attention. As exogenous cueing is suppressed outside spatial attention (Theeuwes, 1991; Ruthruff & Gaspelin, 2018), 1of1 performance should be reduced for narrow attention, approximating that in the 1ofN Condition for perfectly serial attention.

A limitation of the 1of1 Condition was that in presenting only one item, comparisons with NofN and 1ofN Conditions introduced a confound similar to conventional set-size manipulations. I return to discuss this, later. Briefly, however, any potential effects of stimulus interactions (that might benefit 1of1 performance relative to 1ofN or NofN) will either be *dependent* on the breadth of attention in the search task or *independent* of it. Attention-*dependent* effects were not a particular concern as they formed part of the effects of spatial attention that these measures were intended to index. Attention-*independent* effects, by contrast, would threaten the comparison of 1of1 with other conditions, if substantial. However, these latter effects could be assessed in comparisons of 1of1 and 1ofN Conditions under *narrow attention:* to anticipate, no evidence for such effects was found.

**Fig. 3:**
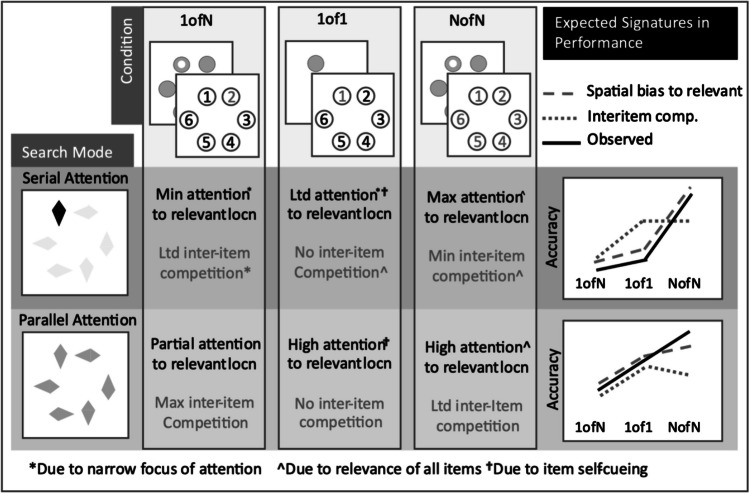
Initial predictions for memory task performance on the basis of spatial attention bias and inter-item competition, under serial or parallel attention. Also illustrated, patterns of task performance observed here. Top row, serial (narrow, sequential) attention, bottom row, under broad, parallel attention. Middle three columns highlight, for 1ofN, 1of1 and NofN Conditions, expected effects of interference/competition among memory items plus degree of spatial attention bias toward relevant information. To anticipate findings here, results from Experiments 1a–4b conformed approximately to predictions of spatial attention patterns for Serial Attention, but for Parallel Attention, underestimated NofN performance relative to 1 of 1 performance.

Figure 3 summarises patterns of memory performance expected for narrow or broad attention in these three primary conditions. For narrow, serial attention, Memory Task accuracy should be high for the NofN (peak attention) Condition, but poor (and similarly so) for 1ofN and 1of1 Conditions, as attention would only rarely be focused at the single, reportable item’s location (approx. 1/N, if 1 item attended) and cueing by the 1of1 item should be minimised. Plotted in order 1ofN (low accuracy), 1of1 (low accuracy), NofN (high accuracy), they should form a deep *downward*-elbow shape (see upper-right-panel, Figure 3). To anticipate, while narrow attention did yield a downward elbow shape. For broad, parallel attention, 1of1 accuracy was expected to approach or equal that in the NofN condition and 1ofN performance below those two Conditions. Plotted in order 1ofN, 1of1, NofN, they were initially expected to form an *upward*-elbow shape (see lower-right-panel, Fig. 3), (presumed) broad attention yielded no elbow (rather than an upward elbow); 1of1 accuracy lay between that for NofN and 1ofN.

Nonetheless, this approach effectively distinguished between broad and narrow attention. This was in contrast to differences between 1ofN and NofN accuracy, which had been expected to be greater for narrow than broad attention. They did not emerge, simplifying analyses and interpretation, but certainly was not expected. The potential reasons for this unexpected result and its lessons for future research adopting ATLAS are discussed in a section of the General discussion.

The two signatures for broad and narrow attention required comparison of performance across conditions. Performance on any individual condition would reflect a combination of spatial attention effects and other factors (e.g., differential cognitive or perceptual load imposed by different search tasks). However, as all three conditions should share those other factors in common, but differ in expected effects of spatial attention, comparing, for example, 1of1 performance to the reference provided by the other NofN or 1ofN conditions should index spatial attention differences between search tasks.

### Pairwise attention estimator: 3ofN:Alt, 3ofN:Adj

**Fig. 4:**
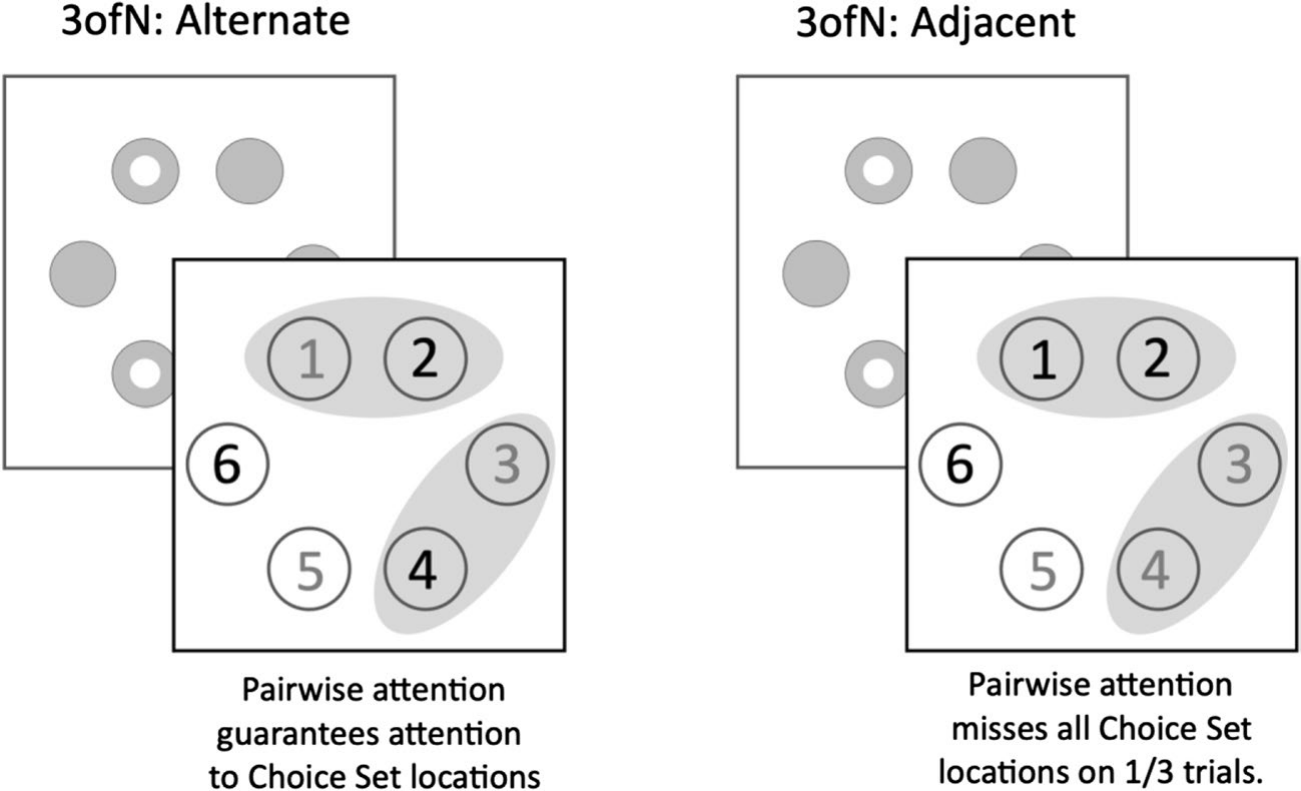
‘Pairwise Attention Indicator’ Conditions, each of which comprised three Choice Set locations (indicated by bold, black numbers). Two possible attention windows selecting pairs of items, are cartooned for each condition as grey ellipses. In the 3ofN:Alt Condition (left), pairwise attention to any two items would guarantee attention to a reportable item from the Choice Set. However, in the 3ofN:Adj Condition (right), pairwise attention would result in no attention to any reportable item on one out of three trials

Two supplementary conditions were included to refine estimates of attention breadth. Both ‘3ofN’ Conditions (Fig. 4) had three reportable items (indicated by green numbers in the Choice Display) but varied in terms of their spatial arrangement. In the ‘3ofN:Alt’ Condition (Fig. 4, left), alternate numbers/locations were green (i.e., reportable – either all odd numbers or all even numbers). Hence, if participants’ attention selected a pair of neighbouring items at once, they would always be attending to a reportable item and performance should approach NofN Performance. In the ‘3ofN:Adj’ Condition (Fig. 4, right), however, all three green numbers were at three adjacent locations. Thus, a participant whose attention selected two items at once would, on 2/N trials (in this case, 2/6) not be selecting any items at green-number locations. Higher 3ofN:Alt than 3ofN:Adj accuracy would therefore be consistent with ‘pairwise’ attention (to items at a time). In contrast, no such advantage should hold for one-at-a-time serial attention, or broad attention to more than three items.

#### Notes on predictions and expected effects

In an unpublished version of this article, observations on earlier versions of Experiments 3 and 4 motivated initial estimation of some effect sizes and expected NofN=1of1 pattern for broad attention. Those two versions (using shared code) were discovered to have stimulus duration miscoded; *the corrected experiments described here* yielded much smaller effect sizes for important between-experiments (Serial vs. Parallel) comparisons here (around d = 0.5, rather than d > 0.8), though fortunately due to replications that had already been included in the article, sufficient for the current analyses. Also please note that target-adjacent ‘dips’ in the attention guidance discussion section later were *not* predicted prior to running these experiments (the author only concluded that they should be expected on local but not global guidance after writing the initial, unpublished manuscript) and crucially they were not observed here. Three experiments, run subsequently to those reported here, do *appear to* exhibit dips (and are part of a larger project applying ATLAS to other search stimuli), but their robustness yet to be confirmed. Relatedly, the plots and discussion clarifying how various guidance modes should influence ordinal attention dynamics were added to the revised article.

## Experiments 1–4: Establishing validity of key ATLAS indicators

 Experiments 1–4 were intended to establish the validity, for a single set of search stimuli, of the key indicators proposed here. No physiological or behavioural measure and no computational model could provide access to the ‘ground truth’ of whether attention is serial or parallel. Instead, to examine the characteristics of ATLAS’s key indicators under serial (narrow, sequential) or parallel (broad, simultaneous) attention, five initial experiments were designed to maximise the intuitive likelihood of stable narrow, serial attention or broad, parallel attention, that could be sampled at a range of SOAs (time lag between Search Display Onset and Memory Display Onset).

Note that, as also addressed in the *General discussion*, the experiments are explicitly focussed on one highly regular, easily controlled set of search stimuli – an example search stimulus selected to elicit clear patterns of attention and processes that would not operate too efficiently (i.e., not support perceptual ‘popout’) and would not encourage perceptual segmentation of the Search Display as many feature-conjunction searches would. Those (and other) complications will each need addressing in order to extend ATLAS’s potential benefits to the gamut of visual search tasks in the literature. The work described here is certainly at the ‘proof of principle’ stage; however, other, subsequent experiments run in the laboratory suggest they apply robustly.

Here, Experiments 1 (1a, 1b) and 2 (2a, 2b) were intended to elicit search with a stable serial attention component (either to one item at a time, in Experiments 1a and 1b, or two items at a time in Experiments 2a and 2b). Experiments 2a and 2b, to encourage attention to two items at a time, required participants to compare pairs of neighbouring search items, so were not intended to be typical or representative of search tasks. Experiment 1 was therefore the primary example of serial search (expected to elicit attention to one, rather than two items at a time).

Experiments 3 (3a, 3b) and 4 (4a, 4b) were instead designed to elicit *stable broad, parallel attention*. This could not be ensured with any *search* task (efficient parallel search *may* elicit broad attention, but only fleetingly so), so those two experiments required participants to monitor (with broad attention) an array of items, not to search. Note, however, for brevity, the same terms ‘Search Task’ and ‘Search Display’ are retained for those two experiments. Experiment 3 was the primary example of ‘Parallel’ search as it used the same search stimulus as Experiment 1 (Experiment 4 used the same shapes, but in a dynamic display). Two further experiments (5a, 5b), described later, applied the indices of attention established in Experiments 1–4 to more typical search tasks and measured attention guidance.

### General methods

#### Participants

Ethical approval was provided by the Department of Psychology Ethics Committee at the University of Cambridge (Ref: 1482/47). In Experiments 1a–4b, 184 participants were recruited via Prolific.co, filtered to specify age 18–35 years and their first language as English. The author attempted to recruit 24 participants for each experiment (20 each for 2a and 2b as insufficient funds were temporarily available). The challenging nature of the online recruitment process rendered it difficult to control sample size precisely and participants were excluded from analysis if they performed below 60% Accuracy on the Memory Task or less than 70% on the Search Task. Actual numbers, combining a and b studies as for most analyses here – Experiment (E)1: 42, E2: 33, E3: 39, E4: 36. The lower cut-off for the Memory Task reflected the fact that in some experiments, low scores were expected for 1ofN and 1of1 Conditions. These studies were not pre-registered; for Experiments and Dataset links, please see *Declarations*. NHST analyses were performed using JASP (Version 0.18 Intel). For the between-participants analyses reported here (unrelated, one-tailed t-tests) the full sample sizes would have provided a priori 85% power to detect an effect of d = 0.4 or 96% for d = 0.5. Reductions in sample size following exclusions meant that the smallest effects reported here had achieved 82% power (G*Power 3.1; Faul et al., 2009). Related t-test effects provided at least 80% power to detect d = 0.45 (a and b versions of being combined; d = 0.55-0.6 in few uncombined analyses).

#### Stimuli and apparatus

**Fig. 5:**
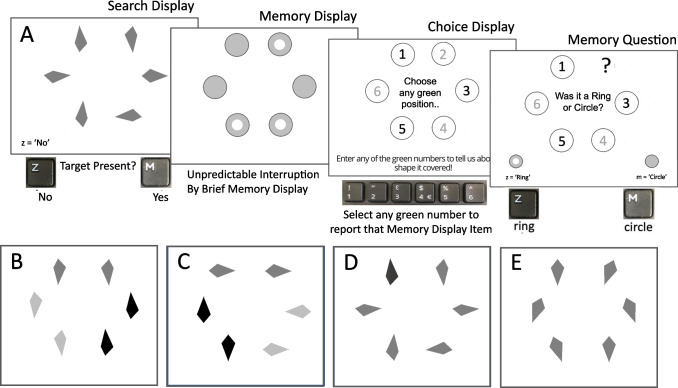
(**A**) Schematises typical sequence of displays in Experiments 1a/1b. Participants searched for kite shapes among lop-sided (asymmetrical) kites. The Search Display either remained until response, or, on 36% of trials, was interrupted by a brief Memory Display, followed by a Choice Display. On these trials, participants selected the to report the Memory Display item they remembered best, by entering the corresponding number from 1-6 (only green numbers could be selected, here indicated in bold, black font). They then reported whether that item was a ring or circle. (**B**) Search Display from Experiment 2a: Participants searched for pairs of matching asymmetrical kite shapes. (**C**) Search Display from Experiment 2b: As for Experiment 2a but now simpler discrimination between pairs of matching and non-matching kite shapes. (**D**) Search Display from Experiments 3a/3b illustrating one shape undergoing a temporary reduction in luminance, for which participants were asked to monitor shapes simultaneously. (**E**) Search Display from Experiment 4a/b: each lozenge shape changed orientation randomly every 300 ms. Participants monitored items for a moment when all were tilted rightward

Participants used their own Desktop PCs or laptops to participate. Stimuli were presented to participants using Gorilla.sc Online Experiment Software. For the Memory Task component shared by all five experiments, three primary displays were presented. First, a Memory Display (50 ms) presented six shapes – two to four of which were grey circles and the rest, rings (Fig. 3, right panel; Fig. 5A for full sequence), evenly spaced around fixation. The single exception was the 1of1 Condition in which only one circle or ring was presented. Then, following a blank, white screen (1,000 ms), the Choice Display was presented. This was always a display of circles labelled with the numbers 1–6. In the 1of1 and 1ofN Conditions, only one number was green (Figs. 2 and 3), and the participant was instructed to ‘choose’ this and to report the Memory Display item presented there. In contrast, for the NofN Condition, all the numbers were green, so participants could choose any of them to report the Memory Display item they best remembered. In 3ofN (alternative and adjacent) conditions, three of the six circles were labelled with green numbers (Fig. 4) indicating that any of those three items could be selected for response. The participant selected a number (and hence, a Memory Display stimulus; see Fig. 5A for an example) by entering a number from one to six on their keyboard. The Response Display then prompted the participant to indicate whether a ring (response key: ‘Z’) or a circle (response key: ‘M’) had been presented at their selected location (Fig. 5A). Even for 1ofN and 1of1 Conditions, for which there was only one option open to the participant, they were still required to enter that number.

Note, the stimuli employed in the ‘Rings’ Memory Task here were explicitly designed to allow a participant with an expected VSTM capacity of three to four items, to code a six-item display (by noting positions of rings, knowing the rest must be circles). Second, the stimuli minimised influences of acuity (the author subjectively assessed this by fixating at locations opposite probed items). Third, the use of a separate Memory Display to probe attention in the Search Display was intended to maximally disengage participants’ attention from search stimuli, minimising influences of search-stimulus perceptual-load effects.

#### General procedure

Following detailed instructions, each experiment trained participants first on the Memory Task alone. For the first block of 10 practice trials, the Memory Display was presented for 500 ms, before a second block of 15 trials in which it was presented for 50 ms, as it would be throughout the rest of the experiment. Separate detailed instructions then followed for the search task (these differed for each study and are described separately, later). Practice on the search task consisted of 22 trials, followed by 18 trials in which Search and Memory tasks were practised together. After a rest period (at least 30 s, then when the participant chose to continue), there were 6 blocks of 56 trials, 216 of which were Search Task trials that were never interrupted. The remaining 120 trials were divided equally between 1ofN, NofN, 1of1 and the two 3ofN Conditions. In these 120 trials the search display was equally likely to be interrupted (if not completed) after 1,000, 1,400 or 1,800 ms, other than for Experiment 3 (a search that could be completed more rapidly, so 800, 1,100 and 1,400 ms). Note that on trials where search was scheduled to be interrupted but in which the participant responded before the interruption (possible in Serial Experiments only), no response or choice to the Memory Display was recorded, as it was not presented (only a response to the Search Display). This typically affected only around 18% trials per condition.

#### Individual search tasks

1. Experiments 1a and 1b, ‘rotated kites’

Participants began every trial of the main experiment by performing a search task. Figure 5A schematises a typical sequence of displays in a trial from Experiments 1a and 1b (Experiment 1b was a replication of Experiment 1a). Following a fixation cross and task cue (“Is there a kite?”) the Search Display was presented (see leftmost panel). Six dark blue shapes appeared and participants searched for (horizontally or vertically symmetrical) kite targets that were presented, unpredictably on half of trials, among ‘lop-sided’ kite nontargets. They pressed the ‘M’ key to indicate that a target was present, the ‘Z’ key to indicate that no target was present. Given the subtle distinction between targets and nontargets, search was anticipated to comprise a strong serial (sequential, narrow attention) component. On 120 of the 336 trials (arranged in five blocks of 56 trials), the Search Display would disappear and be replaced by a Memory Display (after 1,000, 1,400 or 1,800 ms) signalling that the participant should switch to performing the Memory Task. Search Task errors were signalled by the message “INCORRECT: please take care with the search task” in red, for 5 s, Memory Task errors by “Incorrect” in black, for 500 ms.

2. Experiments 2a and 2b: ‘Kite Pairs – Hard’ and ‘kite Pairs – Easy’

Experiment 2a (Kite Pairs – Hard) presented only lop-sided kite shapes, this time segmented into three neighbouring pairs of kites on the basis of colour (see Fig. 5B; one blue pair, one black, one red, as illustrated). Each pair of neighbouring kite shapes were mirror reflections of each other, other than, unpredictably on half of trials, one pair of shapes that were identical to each other. The participant’s task, cued at fixation on each trial (“Is there a matching pair?”), was to determine whether there was a matching pair of kite shapes, or not. In all other respects, the task followed the procedure for Experiment 1. As with Experiments 1a/b, on 120 out of 336 trials, the Search Display was interrupted by presentation of the Memory Display, then the Choice Display. It was intended that this task would encourage attention to neighbouring pairs of items, but on reflection (and the author's subjective impressions, performing the task) this task was likely so challenging that it demanded serial attention to each item (as discussed later).

Experiment 2b (Kite Pairs – Easy) replicated the conditions of Experiment 2a but with a much more salient distinction between matching and non-matching pairs of shapes, to permit comparison of Search Display item pairs without individual attention to each item in turn (i.e., attention to both items simultaneously). Now, all items were kite shapes (not lop-sided) and pairs of them differed from each other by 180° (see Fig. 5C for examples). On half of trials, unpredictably, one of the pairs of shapes were identical to each other. To account for reduced task difficulty, the Memory Display now interrupted the Search Display after 800, 1,100 or 1,400 ms as piloting indicated search could often terminate prior to 1,500 ms.

3. Experiments 3a and 3b: ‘Parallel Monitoring – Luminance’

Experiments 3a and 3b (3b replicated 3a) employed the same stimuli, response keys and Memory Task as Experiments 1a and 1b. However, they were intended to encourage parallel-attention (rather than being a search task). Following a fixation cross and task cue (“Monitor kites for change”), the participant’s task was to monitor all these shapes, simultaneously in parallel, for a luminance decrement applied to one of them (on half of trials, for 200 ms between 400 and 1,600 ms post Search Display onset (Fig. 5D). The magnitude of the luminance reduction could not be controlled precisely in an online experiment but was subjectively calibrated on the author’s own screen to demand attention yet not be sub-threshold. On standard search trials, the Search Display terminated after 2 s and the participant then indicated either that one of the shapes had changed luminance (by pressing the ‘M’ key) or that none had (by pressing the ‘Z’ key). Memory Display interruptions now only arose on No-Change trials to ensure continued broad attention to the end of the task.

#### Experiments 4a and 4b: ‘Parallel Monitoring – Orientation’

Experiments 4a and 4b resembled Experiments 3a and 3b, in that a standard memory task was again combined with a parallel item-monitoring task intended to encourage broad attention. The ‘Search Display’ items were again kite shapes from earlier experiments (Fig. 5E), but each kite now adopted a new orientation (from the same set of three orientations) every 300 ms during an overall display duration of 2,100 ms (six changes, in all). In half of trials, during one 300-ms period (between 600–900 ms, 900–1,200 ms and 1,200–1,500 ms post-onset) all bars would be right-tilted. Preceding each search display, the task cue “Are all kites right-tilted at the same time?” was presented. Following the Search Display, the participant was prompted to respond by the text "Were all lines right-tilted at the same time?” pressing the ‘M’ key to indicate ‘yes’, the ‘Z’ key to indicate ‘no’ when the display ceased to change. Experiment 4b was not a replication of 4a as when not vertically oriented, the kites tilted left or right by 15° in Experiment 3a, 30° in Experiment 4b.

In any monitoring task such as this, variations in temporal attention due to evolving conditional probabilities of a target (given none has been detected at a particular time) cannot be excluded (Lawrence & Klein, 2013; Nobre & Van Ede, 2018). Fortunately, this potential feature of monitoring tasks is also a likely characteristic of inefficient search processes (e.g., when searching serially, the chance that a target is present among the items not yet searched changes over time), so while this remains a limitation, it is perhaps desirable, being shared by Experiments 1–3. Note, monitoring tasks were chosen here to elicit *stable* broad spatial attention over time. ATLAS might subsequently also be used to examine very fleeting parallel attention processes in popout, but in this initial project, the priority was to demonstrate ATLAS Condition signatures under stable conditions.

For brevity and to optimise power, ‘a’ and ‘b’ versions experiments were merged for analysis and report. Initial analysis of the ‘Serial’ Experiments 1 and 2 confirmed that the expected patterns were broadly observed for the three primary conditions (1of1 accuracy poorer than NofN and similar to 1ofN accuracy) *and* that the pattern was very similar in Experiment 2 versus in Experiment 1. Accordingly, Experiments 1 and 2 were then combined to optimise power when comparing these primary conditions under narrow, serial attention versus broad, parallel attention in Experiments 3 and 4. The primary comparisons were, for each index, an unrelated t-test to compare scores from Serial and Parallel experiments. Note that – *while this decision did not impact any of the outcomes described* – each between-experiments t-test reported here is one-tailed as each could only feasibly be predicted (and interpreted) in one direction. There is justifiable mistrust of using such tests, but their use in ATLAS for future work will maximise power.

As indicated earlier, a further prediction was that a third subtraction (NofN-1ofN Index) should yield greater scores for narrow than broad attention experiments. This had not been considered the primary index (as scores were not expected to approach zero under any circumstances) and, here, did *not* differ between broad and narrow attention. This unexpected finding is addressed in the *Results and discussion* section.

### Experiments 1a–4b: Results

Mean RT and Accuracy for ‘Serial’ Search Tasks in Experiments 1a–2b were (mean (SEM)): E1a: RT, Target Present (TP) = 2,533 ms (230 ms), Target Absent (TA) = 3,485 ms (335 ms), Accuracy = 89% (1.6%), E1b: TP = 2,062 ms (184 ms), TA = 2,827 ms (255 ms), Accuracy = 88% (1.6%), E2a: TP = 2,558 ms (390 ms), TA, = 3,260 ms (400 ms), Accuracy = 91% (1.8%), E2b: TP = 1,549 ms (117 ms), TA = 2,115 ms (156 ms), Accuracy = 94% (1.0%). For the two Parallel Experiments, there were no RTs. Accuracy, E3 = 89% (1.2%) and E5: = 96% (0.7%). These were not analysed further.

**Fig. 6:**
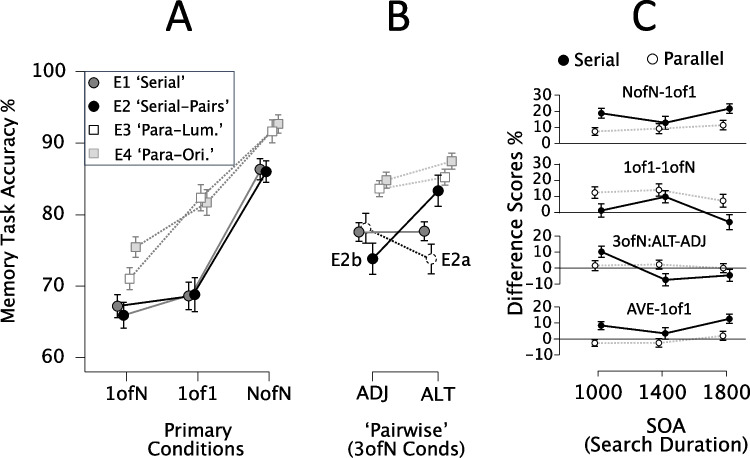
(**A**) Memory Task Accuracy for primary conditions (1ofN, 1of1, NofN) in Experiments 1–4. For Serial Experiments 1 and 2 (1a, 1b, 2a, 2b) 1of1 accuracy was similar to 1ofN accuracy, but for Parallel Experiments 3 and 4 (3a, 3b, 4a, 4b) 1of1 accuracy lay halfway between NofN and 1ofN performance. (**B**) as for A, but 3ofN:ADJ and 3ofN:ALT conditions (E2a and 2b plotted separately reflecting presumed pairwise attention difference; other scores very similar so analysed together. (**C**) Indicator scores across matched stimulus onset asynchronies (SOAs) for replicated Serial and Parallel experiments (E1, E3). NofN-1of1 scores were smaller for Parallel than Serial experiments, 1of1-1ofN scores were larger; scores were particularly stable for presumed parallel attention. Pairwise Attention (3ofN:ALT-ADJ) scores remained stably around zero for the Parallel experiment, but consistent with pairwise-to-serial shifts across SOAs in Serial experiment (present in both E1a and E1b). ‘AVE-1of1’ scores were around zero for Parallel Experiment, above that for the Serial Experiment

Figure 6 plots performance for the Memory Task Conditions. Figure 6a shows mean accuracy for the three Primary Conditions of each experiment: 1ofN, 1of1 and NofN. Note, for ‘Serial’ (narrow, serial or pairwise attention expected) Experiments (1 and 2), these data formed a clear downward elbow (greater NofN accuracy than either of the other conditions), as predicted. In contrast, ‘Parallel’ (broad attention expected) Experiments (3 and 4), 1of1 accuracy lay approximately midway between 1ofN and NofN performance. This did not conform to predictions for perfectly broad attention but did distinguish broad from narrow attention (‘Serial’ from ‘Parallel’) experiments. The deviation from prediction may have reflected sustained attention to only three or four Search Display (and hence, Memory Display) items in those latter tasks, though other explanations could not be fully excluded. Figure 6b panel plots Memory Task Accuracy for the 3ofN:Alt and 3ofN:Adj Conditions, for the same experiments (N.B.: plotted separately for Experiments 2a vs. 2b), where these scores, but not the primary conditions, were expected to differ (see *Methods*). Note the marked (and expected) advantage for 3ofN:Alt over 3ofN:Adj accuracy for Experiment 2B, presumably reflecting pairwise attention there. This strong effect was not generally found for experiments with narrower or broader attention.

A first section of results, below, addresses each the three primary predictions above and a second section provides supporting plots and analyses. Parametric tests and effect sizes are reported here (Welch’s test, where appropriate). Each participant’s 1of1 score was subtracted from their NofN score to yield difference scores (NofN-1of1). These were, as expected, greater for ‘Serial’ (Experiments 1 and 2) than ‘Parallel’ experiments (Experiments 3 and 4; t(148) = 2.908, *P *= 0.002, d = 0.475). While these did not approximate zero in either case (so did not provide an obvious ‘marker’ for serial or parallel attention with the current stimuli), they nonetheless were *clearly* different for broad versus narrow attention. 1of1-1ofN scores were also greater, overall, for Parallel than Serial Experiments (t(148) = 2.551, *P* = 0.006, d = 0.417). These scores exceeded zero for the ‘Parallel’ Experiments (E3: t(38) = 4.634, *P *< 0.0001, d = 0.742, E4: t(35) = 2.583, *P *= 0.007, d = 0.43), whereas no evidence was found that they exceeded zero for the ‘Serial’ Experiments (E1: t(41) = 0.545, *P* = 0.294, d = 0.084, E2: t(32) = 0.901, *P* = 0.187, d = 0.157).

These comparisons of the 1of1 accuracy with 1ofN or NofN Conditions suffered, of course, from a stimulus confound. NofN and 1ofN Memory Displays comprised six items (vs. one in the 1of1 Displays) so performance in those conditions may have been influenced by inter-item interference and competition, that was absent in the 1of1 Condition. To the extent that these influences that were spatial-attention dependent, and hence correlated strongly with spatial attention, they can be considered part of indexed spatial attention. However, any such influences that were *attention-independent* would be nuisance variables, obscuring indexing of spatial attention. Any such attention-independent stimulus effects should have been evident equally in Serial Experiments and Parallel Experiments, whereas attention-dependent effects should only (or primarily) have arisen in the Parallel Experiments. As no evidence of differences between 1of1 and 1ofN performance was found in the Serial Experiments, only in the Parallel Experiments, it appears that no substantial attention-independent stimulus-based effects arose here.

NofN-1ofN difference scores were calculated as for the other indices above. Contrary to expectations, no evidence was found that they differed between ‘Serial’ and ‘Parallel’ Experiments (t(148) = -0.288, *P *= 0.387, d = 0.047). These effects were not analysed further. However, this contradiction of expected findings is addressed in the discussion section that follows. Finally, to establish a ‘Pairwise Attention Indicator’ difference scores subtracting 3ofN:Adj scores were subtracted from and 3ofN:Alt scores. No overall Serial versus Parallel comparison was conducted as Pairwise Attention was only expected, overall, in Experiment 2. As anticipated in the *Methods* section, no evidence was found in Experiment 2a for such a pattern (t(16) = -1.843, *P *= 0.918, d = 0.447, any trend in the opposite direction). The author subsequently concluded that the great difficulty of inter-item comparison had ensured serial, one-at-a-time attention there, not pairwise attention, and designed Experiment 2b to address this. Experiment 2b (‘Kite Pairs, Easy’) confirmed that, as predicted for pairwise attention 3ofN:Alt scores were greater than 3ofN:Adj scores (t(16) = 3.081, *P *= 0.004, d = 0.747); this is apparent when viewing the plot in Fig. 6, centre panel). Other experiments did not show this difference E1:t(41) = 0.054, *P *= 0.478, d = 0.008; E3: t(38) = 1.009, *P *= 0.319, d = 0.162, E4: t(34) = 1.661, *P *= 0.053, d = 0.277), as predicted. These findings supported the idea that the 3ofN:Alt-3ofN:Adj subtraction had indexed attention to neighbouring pairs of items. The unclear, potentially-reversed effects for Experiment 2a are discussed later.

#### An index for broad attention that approximates zero

NofN-1of1 scores were greater for narrow than broad attention but did not provide a clear benchmark expected value for broad attention (as 1of1-1ofN scores had done, for narrow attention). However, they did not establish whether, for narrow attention, but not broad attention, 1of1 scores were *nearer to* 1oN scores than to NofN scores. To do so, an *unplanned* analysis calculated scores that subtracted each participant’s 1of1 accuracy score from the mean of scores for NofN and 1ofN conditions (‘Ave-1of1’): narrow attention would yield positive scores and broad attention, here, scores around zero. While unplanned analysis should be treated with caution, this simple extension served to illustrate its potential use in future studies. These Ave-1of1 scores differed between Serial or Parallel Experiments, as expected (t(148) = 2.933, *P *= 0.002, d = 0.479); there was evidence that they exceeded zero for both Serial Experiments (E1: t(41) = 3.453, *P *= 0.0007, d = 0.533, E2 t(32) = 2.446, *P *= 0.01 d = 0.426), but no evidence that they did so for either Parallel Experiment (E3: t(38) = -0.450, *P* = 0.672, d = 0.045, E2 t(32) = 1.081, *P* = 0.144, d = 0.180).

#### Tracking attention breadth across SOAs

Experiments 1 and 3 were the primary examples of ‘Serial’ and ‘Parallel’ Experiments here, comprising the same SOAs and each including a replication (as opposed to Experiments 2 and 4). Accordingly, they were used to map the performance indices here over across SOAs. These are plotted in Fig. 6c. Note that the repeated-measures ANOVA with a within-participants factor of SOA (1,000, 1,400, 1,800) and between-participants factors of Experiment (Serial E1, Parallel E3) and Version (Original, Replication), yielded a main effect of Experiment (F(1,77) = 5.377, *P *= 0.023, η_p_^2^ = 0.065), but no other main effects or interactions (max F(1.92,148.176) = 1.853, *P *= 0.160, η_p_^2^ = 0.024). This was consistent with a general difference in NofN-1of1 scores between serial and parallel attention tasks that did not vary markedly as a function of SOA. 1of1-1ofN scores similarly differed for the Parallel from Serial Experiment again F(1,77) = 7.009, *P *= 0.01, η_p_^2^ = 0.083), yielding no other main effects or interactions (max F(1.867,143.759) = 0.957, *P *= 0.381, η_p_^2^ = 0.012), with one exception: these scores independently varied by SOA (F(1.867,143.759) = 5.376, *P *= 0.007, η_p_^2^ = 0.065), decreasing on average at the longest SOA.

The further metric illustrated here, ‘Ave-1of1’ scores, yielded parallel findings to these – main effects of Experiment (F(1,77) = 7.748, *P *= 0.007, η_p_^2^ = 0.091) and of SOA (F(1.966,151.354) = 3.815, *P *= 0.025, η_p_^2^ = 0.047), but no other main effects or interactions (max F(1.966,151.354) = 0.965, *P *= 0.382, η_p_^2^ = 0.012). These latter findings, too, were consistent with a broadly stable difference between the Serial and Parallel tasks that did not differ as a function of Version (Original, Replication) or SOA (effectively, processing time, between 1,000 and 1,800 ms).

The final analysis, of Pairwise Attention Indicator conditions, however, yielded a different pattern: no main effect of Experiment (Serial, Parallel; F(1,77) = 0.378, *P *= 0.541, η_p_^2^ = 0.005)), but a main effect of SOA (F(2,154) = 4.616, *P *= 0.011, η_p_^2^ = 0.057) and an interaction between Experiment and SOA (F(2,154) = 3.979, *P *= 0.021, η_p_^2^ = 0.049). There were no other main effects or interactions (max F(2,154) = 0.915, *P *= 0.403, η_p_^2^ = 0.012). The interaction between SOA and Experiment reflected relatively stable scores in the the Parallel Experiment (F(2,154) = 0.182, *P *= 0.834) staying around zero, but changing across SOAs in the Serial Experiment (F(2,154) = 6.809, *P *= 0.002). For the Serial Experiment, scores at the shortest SOA suggested pairwise attention (resembling across-SOA means for Experiment 2b; t(41) = 3.363, *P *= 0.002, d = 0.519), but any tendency was reversed for the two longer SOAs (resembling across-SOA means for Experiment 2a). For comparison, scores for Experiment 2b were positively biased (and increasingly so) at all SOAs (6%, 8%, 14%), whereas changes in scores for Experiment 2a (+2%, -11%, -12%) paralleled those for the Experiments 1a and 1b.

### Discussion

Experiments 1a–4b established key indices of attention breadth: NofN-1of1 (Parallel/Serial Indicator) scores, 3ofN:Alt-3ofN:Adj (Pairwise Attention Indicator) and, relatedly, AVE-1of1 scores. Broad attention was associated with AVE-1of1 scores of around zero and, relatedly, positive 1of1-1ofN scores. Narrow attention was associated with 1of1-1ofN scores around zero and, relatedly, positive AVE-1of1 scores. Where narrow attention was paid to 2 (or perhaps 3) items at a time, this was associated with positive 3ofN:Alt-3ofN:Adj scores.

What of the NofN-1ofN scores that did not differ between broad and narrow attention, but were expected to do so? While this might be viewed as convenient, simplifying comparison of combinations of NofN-1of1 and 1of1-1ofN scores across experiments, it was puzzling.

These limitations aside, Experiments 1a–4b provided some reassurance that ATLAS can provide support for estimates of attention breadth in search experiments. To help distinguish the five modes of search outlined in the Introduction, ATLAS was also required to distinguish possible modes of attention *guidance* by parallel processing. The next section discusses this, supported by two further experiments, using the same stimuli as for previous experiments, but using a search task that encouraged guidance of attention.

## Using ATLAS to reveal attention guidance during search

ATLAS tracked attention guidance in Experiments 5a and 5b by examining the *positions* of the Memory Display items that participants chose to report in the NofN Condition and their relationship to the position of the target item in the Search Display. This indexed the degree of attention bias with respect to the target (hereon, ‘Target Bias’) during search. Increases in target bias across SOAs are to be expected on most of the five search modes considered here, but the expected shapes of target bias across time differ between modes. Note that these target bias estimates are not necessarily related directly to densities of RTs at particular SOAs, though these relationships might usefully be studied in future computational-modelling efforts.

### Patterns of developing target bias

**Fig. 7:**
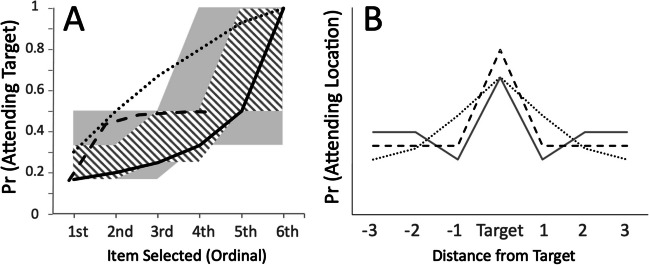
(**A**) Plots expected likelihood of attending (and choosing in the NofN Condition) for serial search processes selecting one item at a time (solid line), to pairs of neighbouring items (region shaded with right-tilted lines: bottom edge = no local guidance, top edge = maximum local guidance), three neighbouring items at a time (grey region, bottom edge = no local guidance, top edge = maximum local guidance). The dotted black line indicates a typical expected pattern for constant, global guidance (see text). The black, dashed line cartoons pattern observed in Experiments 5a and 5b, consistent with either attention to three items plus local guidance, or with imperfect, global guidance that initially asymptotes at a Target Bias of 0.5. These two possibilities can be distinguished by the presence of ‘dips’: see text. (**B**) Expected spatial distribution of NofN choices for serial attention with precise global guidance (dashed line), with imprecise global guidance (dotted line) and for pairwise attention with local guidance (grey line; note target adjacent ‘dips’, see text)

How should target bias be expected to emerge across SOAs for the five modes? Let us consider, first, examples of ‘SERIALnone’ (unguided, sequential application of narrow attention). The simplest example of this is classic serial, unguided search to exactly one item at a time. Let us assume, for simplicity, that attention never repeatedly selects an item. That likely won’t be so, but any tendency to perseverate would further flatten the curves plotted here, making them *easier* to discriminate from guided attention. Being unguided, attention will be equally likely to select any of the six Search Display locations first, so the probability that the first item selected by attention is the target (and hence, that the *target’s location is chosen*) is 1/6 (0.1667). If that item is not the target, the probability that the next selected item is the target is 1/5 (0.2; drawn from remaining unsearched items), then 0.25, 0.333, 0.5 and 1. Note, these odds (plotted in Fig. 7 as a thick, continuous line, for ordinal attention – first to sixth item selected – *not* time per se, as that is poorly constrained) increase *slowly* for the first half of items searched, then more rapidly.

If, instead, attention selects ‘clumps’ of two or three items without any guidance (i.e., still the ‘SERIALnone’ mode) fairly similar patterns should emerge. In Fig. 7a, the *bottom edges* of the diagonal-line and gray shaded regions plot expected patterns for attention to two- or three- item clumps, respectively. These probabilities are derived by assuming that if a clump of, for example, three items is attended, each is roughly equally likely to be chosen if search is interrupted. Note that, in the absence of parallel guidance mechanisms, the lower edges of the shaded regions approximately track those for serial, one-item-at-a-time search, for the first four items searched.

The *upper* edges of the same shaded regions plot, for unguided selection of two- and three-item clumps, the maximum likelihood of attending the target if *there is local guidance* within each attended clump of items (‘SERIALlocal’ mode). This local guidance would increase the probability that the target, if present within the attended clump, is selected. Highly efficient guidance might always ensure the target is always attended first, if present, but the effect might accrue more slowly, if less efficient. Accordingly, this local guidance might mimic the effects of global guidance on target bias.

A pattern for narrow attention *guided by steady global parallel processing* (SERIALglob) could take a large variety of different forms. To illustrate one such form (and thereby the logic of expecting this form), one potential curve is cartooned as a dotted line in Fig. 7a. Compared to the prediction for unguided, serial selection of one item at a time (solid line), the likelihood of selecting the target first in this cartooned example, is greater than chance and this bias will only increase as the second and third items are selected such that attention may only have to select three to four items in order to ensure the target is found on nearly 100% of trials. The expectation here is that if guidance is highly efficient it should always ensure that the target is the first attended item, but that for weaker or noisier guidance, the target won’t always be attended first, but will likely be one of the first one or two items attended. Still weaker guidance will bias attention only slightly toward the target. Broadly speaking, however, *positive curvature (steep initial increase, then asymptote) will be consistent with guidance, inconsistent with unguided search*, and the degree of curvature is expected to vary as a function of the strength/rapidity of guidance.

To anticipate findings from Experiments 5a and 5b, some examples of SERIALglob may yield pattern like that of the dashed line; that is, with positive curvature indicating guidance, but asymptoting initially around 0.5, not 1. This should be expected when attention is guided by global parallel processing but not uniquely toward the target. For example, if the target is red ‘O’ among two red ‘Q’ nontargets and three orange ‘O’ nontargets, attention will likely be guided rapidly to one of the red items by global parallel processing of colour, but not necessarily toward the target. Rather, it might be that attention is guided randomly in a first wave of guidance either to the target or toward the two red nontargets. In such a case, the probability that attention selects the target’s location will rise rapidly from chance (1/6) to 1/3 (as the target is one of three red items), but might then not increase rapidly, as serial search begins (guidance becoming less efficient once attention has narrowed onto the first selected item). Global guidance may therefore, in these respects, mimic local guidance.

The two other search modes involved broad, simultaneous attention. PARAnone, simultaneous, parallel attention to all items, with no guidance would predict constant probability of choosing the target’s location of 1/6 across SOAs. PARAglob: simultaneous, parallel attention to all items, with global guidance toward the target, would instead predict a similar pattern to that of narrow attention with global guidance; indeed, this mode, PARAglob would be distinguishable from SERIALglob only in terms of the measured breadth of attention during earlier stages of search.

### Target-adjacent ‘dips’ in NofN selections distinguish local- from global guidance

In the previous section, it was noted that local guidance (in SERIALlocal search) and global guidance (in SERIALglob or PARAglob) may mimic one another in terms of expected initial development of target bias. However, ‘dips’ in a pattern in NofN choices should help to distinguish the two types of grouping. To illustrate, consider Fig. 7b, which plots characteristic distributions of expected locations, relative to a Search Target’s location, that participants will select for report in the NofN Condition. Along the x-axis is location, where the central data points plot the proportion of times the Target’s location will be selected, those at -1 and +1 plotting locations adjacent to the target (anticlockwise and clockwise, respectively), -2 and +2 the items adjacent to those, and 3 the item opposite the target’s location. Note, there are no specific values on the y-axis: the shape of the distribution is primary; the only constraint is that the six proportions must sum to one.

The dotted line plots an expected pattern for SERIALglob where attention selects clumps of two or three items (or one item, but is imprecisely guided), and the dashed line, where it selects just one item at a time. Attention is most likely to be at the target’s location, sometimes also being more likely at target-adjacent locations (in cases of broad-attention or imprecise guidance). Note that these same distributions may also emerge slowly for serial, unguided attention (as noted above, odds of attending the target increase with each extra item attended, even without guidance). However, for reasons described above with respect to Fig. 7a, this emergence should be slower than is typical for guided search, showing largest increases at SOAs sampling around the longest RTs (certainly above the median) for the same search task (assuming Search Targets attended later will tend to yield longer RTs).

These plots can distinguish global, parallel guidance (SERIALglobal, PARAglobal) of attention from SERIALlocal attention to a clump of items followed by *local* guidance by parallel processing, *but only of items within each attended clump*. Local guidance can, as discussed earlier, increase the likelihood that the target item is selected over time. However, in doing so, it should *decrease* attention, not from locations opposite to the target, but rather from non-target locations *within the clump.* Accordingly, on SERIALlocal, increased choices of the target’s location should be accompanied by decreases of choosing target-adjacent locations. The solid grey line in Fig. 7b plots the expected distribution of NofN choice locations for SERIALloc: unguided attention to *pairs of items* within the search task, followed by guidance to the target (if in the attended pair). This approach is readily extended to attention to three items at a time.

Note, the approach advocated for here does not allow for asymmetry of choices around the target; if that is very marked, it may reflect a tendency to search (anti)clockwise. Further, in principle, local and global guidance may co-occur in search, one counteracting the effect of the other in target adjacent choices. To assess, more formally, whether target bias is only due to local guidance, or not, we can calculate three proportions: *P*_*1*_*, P*_*2*_, *P*_*3.*_

### Three proportions, P_1_, P_2_, P_3_: Does target *bias* exceed attention to one, two or three items?

If attention is selecting just one item at a time (unguided or globally guided) an appropriate measure for this bias is simply the proportion of NofN Choices that are at the target’s location, here ‘*P*_*1*_’. If, instead, attention is selecting two items at a time, *P*_*1*_ may exceed 1/6 due to local guidance alone, but in such a case, *P*_*2*_, the *mean* proportions of choices of the target and the adjacent location either side (i.e., three locations in all); the expected value for target bias only due to local guidance is again 1/6). For attention to three items at a time, the corresponding value, *P*_*3*,_ is calculated as the mean proportion of choices of the target location and *two* locations either side; this also should not exceed 1/6 on the basis of local guidance alone.

## Experiments 5a and 5B: Tracking attention guidance

Two final studies, Experiments 5A and 5B, were designed to provide a practical example of these constraints, using the same stimuli as in Experiments 1–4, but now encouraging and indexing attention guidance. Experiments 5a and 5b employed the same Search Displays as the Target-Present Displays of Experiment 1: each comprised one kite shape at one of four possible angles, among five other lop-sided kite shapes, again at four different angles. Prior to each search display, a search instruction now informed participants as to the target’s orientation (if it was present). This was intended to allow participants, by forming a search template for that particular shape’s orientation, to guide their attention toward candidate target items, using *parallel* processing of the kite’s orientation. Experiments 5a and 5b differed only in terms of the Memory Task Conditions included and the range of SOAs sampled (as described below).

### Method

All aspects of the methods were as for Experiment 1a, with the following exceptions. First, a kite shape was always present among lop-sided kites. Second, prior to each Search Display, an Instruction Display (2 s) presented a kite shape (for the first second) at one of four orientations, at fixation, with the question: “Is *this* kite present?” and in parenthesis, “pointing upward” (or downward, leftward, rightward, as applicable) just above it. If the kite shape in the subsequent Search Display was at the same angle as the kite in the Instruction Display, it was the target and the participant indicated ‘target present’ by pressing ‘M’. Otherwise, they pressed the ‘Z’ key to indicate ‘target absent’.

Some adjustments were also made to the Memory Task Conditions. In Experiment 5a, only 1of1, 1ofN and NofN Conditions were included (1ofN and 1of1, each 36 trials, plus NofN, 54 trials to maximise sensitivity when establishing this component of the methods) and all ATLAS (interrupted search) trials now in Target-Present Search Displays. This adjustment meant that NofN scores might not be comparable between Experiments 5a/5b and the other experiments; this was accepted as the primary function of Experiments 5a and 5b was to examine guidance measures. There were 240 uninterrupted search trials, plus the same practice trials as Experiments 1a and 1b. SOAs were 700, 1,000 and 1,300 ms. In Experiment 5b, 48 Pairwise Attention Indicator trials (24 3ofN:Alt, 24 3ofN:Adj) replaced the 1ofN trials and SOAs were now: 100 ms, 400 ms and 1,000 ms. While limiting the overall duration of the experiment, not all combinations of the Pairwise-Attention Indicator trials (Search Target Position × Probe Position × SOA) could be equalised: these minor inhomogeneities were counterbalanced across participants. A minor coding error involving four trials, meant that trials on which the Target and Probed position matched in the 1ofN Condition were not evenly spread across SOAs. However, this did not impact any of the analyses reported here.

#### Participants

Twenty-four participants were recruited for each experiment. In Experiment 5a, five participants scored below 60% on Memory Task accuracy (and similarly low on Search Task Accuracy) and so were excluded. In Experiment 5b, one participant scored below chance on the Memory Task, a second, below 60% on Search Task accuracy was (below 70% cutoff) and one participant’s data were not successfully recorded.

### Results and discussion

Mean RTs and Accuracy for Experiment 5a were: Target Present: 1,563 ms, SEM: 76 ms, Target Absent: 2,074 ms, SEM: 117 ms, Accuracy: 92%, SEM: 1.2%; for Experiment 5b, RT: Target Present: 1,574 ms SEM: 105 ms, Target Absent: 2,031 ms, SEM 131 ms, Accuracy: 87% SEM: 2.5%.

#### Attention breadth measures

**Fig. 8:**
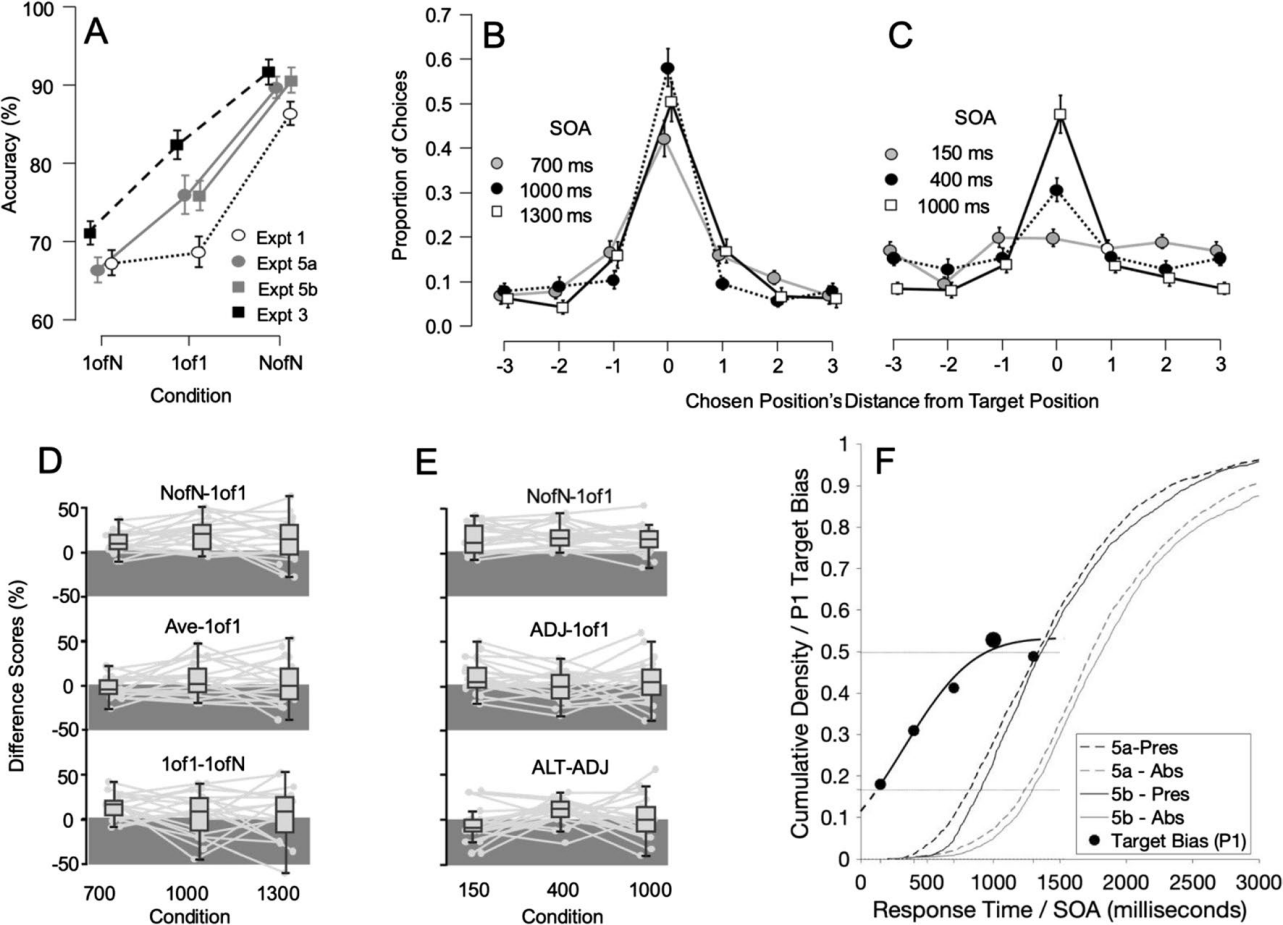
(**A**) 1ofN (5a only), 1of1, NofN accuracy in Experiments 5a and 5b – plus for Experiments 1 and 3, for comparison. (**B**) Proportion of NofN choices in Experiment 5a at Search Target’s location (centre data points) or at points clockwise or anticlockwise (see text), separately for each stimulus onset asynchrony (SOA). (**C**) as B, but for Experiment 5b. (**D**) Variation of key indices across SOAs in Experiment 5a. (**E**) as D, but for Experiment 5b. (**F**) Cumulative density functions for response times (RTs) in Experiments 5a and 5b; on the same plot, Target Bias at various SOAs in those experiments. Note that attention guidance (changes in Target Bias across SOAs) followed the pattern presumed to indicate either global guidance of attention or attention to a clump of three items, plus local guidance. The absence of target-adjacent ‘dips’ (in fact, typically the opposite pattern) is more consistent with the former possibility (see text)

There was no prediction with regard to whether Experiments 5a and 5b would yield broad or narrow attention. Figure 8a plots 1ofN, 1of1 and NofN Scores for Experiment 5a  and, for visual comparison, replots those for Experiments 1 and 3 (‘Serial’ and ‘Parallel’ attention examples, using the same search displays as Experiments 5a and 5b). The pattern of performance across the three primary conditions (1ofN, 1of1, NofN) for Experiment 5A more closely resembled that for Experiment 3 than Experiment 1, consistent with broad attention. NofN-1of1 differences scores for Experiments 5a (and Experiment 5b; see extra pair of data points in Figure 8a) could not alone distinguish Experiments 5a and 5b from either Experiment 1 or Experiment 3 here, as the number of NofN trials was increased (these experiments primarily serving to test ATLAS’s measurement of *guidance* rather than attention breadth). However, 1of1-1ofN differences for Experiment 5a resembled those of Experiment 3, rather than Experiment 1 and were greater from zero ((t(18) = 2.979, *P *= 0.004, d = 0.683) as in Experiment 3, but not Experiment 1). Ave-1of1 scores for Experiment 5a, plotted in Figure 8d, remained around zero for each SOA (t(18) = 0.705, *P *= 0.245, d = 0.162), resembling findings for ‘Parallel’ Experiments and hence consistent with broad attention. To maximise power in future work, indices for a given search task might be compared to the *mean* expected for either serial or parallel search (estimated in separate conditions), rather unrelated t-tests.

In Experiment 5b, the 1ofN Condition was replaced by the two 3ofN (ALT, ADJ) conditions to supplement the NofN-1of1 scores and reveal any tendency to select clumps of two to three items in the search task (common to 5a and 5b). As NofN and 1of1 scores were both near-identical in Experiments 5a versus 5b (Figs. 8a and 8b), we may assume that those attention-breadth indicator scores were similar for the two experiments. To provide an approximation to the Ave-1of1 scores expected, 3ofN:Alt-1of1 scores were calculated and plotted (as 3ofN:ADJ scores also approximated those of the 1of1 Condition in the ‘Parallel’ Experiments). Like the Ave-1of1 scores for Experiment 5a, these scores appeared to remain steadily around zero across SOAs in Experiment 5b (see Figure 8e), consistent with broad attention. Further cross-experiment comparisons were not included as these were not the primary focus of Experiments 5a and 5b.

With regard to potential changes in attention breadth over time (Figs. 8d and e), there was no evidence that NofN-1of1 scores changed reliably across 150 to 1000 ms SOAs in Experiment 5b (one-way ANOVA, with factor of SOA: F(2,40) = 0.532, *P *= 0.591, η^2^ = 0.026), nor across longer SOAs in Experiment 5a (700–1,300 ms, F(2,36) = 2.023, *P *= 0.147, η^2^ = 0.101). This was also true for 1of1-1ofN scores (F(2,40) = 0.532, *P *= 0.591, η^2^ = 0.026) and Ave-1of1 scores (F(2,40) = 0.532, *P *= 0.591, η^2^ = 0.026) in Experiment 5a. However, for Pairwise Attention scores in Experiment 5b (again, one-way rm ANOVA with factor of SOA), the result was less clear (F(2,40) = 2.772, *P *= 0.074, η^2^ = 0.122). Planned t-tests revealed no Pairwise-Effect at 150ms (t(20) = -1.346, *P *= 0.903, d = 0.294), an effect at 400 ms (t(20) = 3.042, *P *= 0.003, d = 0.664), but none at 1,000 ms (t(20) = 0.120, *P *= 0.453, d = 0.026). While this may simply have reflected a Type-1 error at 400 ms, it may also reflect the possibility that typical ‘broad’ attention in the ‘Parallel’ attention experiments (Experiments 3 and 4) may have been to three to four items, rather than six as intended. Indeed, the marginal pairwise-indicator score (while not interpreted as solid evidence for pairwise attention) in Experiment 4 is consistent with attention to three to four items in that experiment (as the pairwise attention indicator should yield a weak signal for attention to three items).

#### Target *bias*

The primary element of interest in Experiments 5a and 5b was attention guidance, and its measurement as changes in Target Bias (the tendency to choose to report the Search Target’s location in the NofN Condition). Figure 8b plots the proportion of NofN Choices by distance from the target (zero being the target’s location, -1 or +1 denoting target-adjacent items, etc.) in Experiment 5a. At each SOA (700, 1,000, 1,300 ms), *P*_*1*_ (proportion of NofN trials in which the target’s location was chosen) as expected, always exceeded 1/6 (across SOAs, min t(18) = 7.090, *P* < 0.0001, d = 1.626). A one-way repeated-measures ANOVA on these bias scores revealed a main effect of SOA (F(2,36) = 8.630, *P *< 0.001, η^2^ = 0.324) – target bias had changed over time. Following an increase in target-bias between 700 and 1,000 ms (t(18) = 3.675, *P *= 0.002, d = 0.843) and then a minor decrease from 1,000 to 1,300 ms (t(18) = -2.142, *P* = 0.046, d = 0.491). This was consistent with a ‘wave’ of attention guidance ending at around 1,000 ms, followed by either initiation of unguided serial search, or initiation of a second wave of guidance to select a second item. Did this target bias reflect unguided attention to a single clump of items, plus local guidance within the attended clump? Likely not: the was no evidence for this: no ‘dips’ evident in proportions of NofN choices and, correspondingly, *P*_*2*_ and *P*_*3*_ also each always exceeded 1/6 (min t(18) = 3.425, *P *= 0.003, d = 0.786), strongly suggesting that local, within-clump guidance was not the sole cause of target bias.

However, these findings did not provide clear evidence for or against target bias accrued via unguided serial (one-at-a-time or clump) attention (without local guidance, see Fig. 7a). To assess that possibility required sampling at earlier SOAs to establish the *shape*, across SOAs of target bias evolution. Figure 8c plots target biases in Experiment 5b, which sampled at much shorter SOAs (same format as Fig. 8b). A one-way repeated-measures ANOVA on *P*_*1*_ Target-Bias Scores revealed an effect of SOA (F(2,38) = 28.721, *P *< 0.0001, η^2^ = 0.590; target bias appeared to increase with SOA. Follow-up t-tests revealed that target bias did not differ from chance (1/6) at 150 ms (t(20) = 1.098, *P* = 0.285, d = 0.240) but did at 400 and 1,000 ms (min t(20) = 5.766, *P *< 0.0001, d = 1.258). Consistent with the absence of large target-adjacent ‘dips’, significant *P*_*2*_ and *P*_*3*_ scores at 1,000 ms (min t(20) = 6.919, *P*<0.0001, d = 1.510) suggested that local guidance played no major role (at 400ms, *P*_*2*_ scores exceeded chance t(20) = 4.492, P<0.0001, d = 0.980, but did not do so for *P*_*3*_: t(20) = 1.586, *P* = 0.128, d = 0.346). To optimise power in future analyses, these tests should be one-tailed as the predicted direction is clear.

Figure 8f plots the cumulative distribution of RTs for Experiments 5a and 5b, plus on the same x-axes, P_1_ target bias score for SOAs sampled in those two Experiments. Note the shape of the distribution of P_1_ scores and its timing relative to the distribution of RTs. These are consistent with global guidance (SERIALglob or PARAglob) or SERIALloc process working on clumps of around three items, but not as expected for a SERIALnone (serial, unguided attention) or PARAnone process. As P_1-_P_3_ score analyses above had not provided evidence of major local (within clump) guidance processes, SERIALloc was also not preferred as an explanation of these findings. Rather, given that attention breadth was concluded likely in the three- to six-item range, the finding of global guidance and broad attention suggest PARAglob as the most likely mode of search in these tasks.

The time-course of target bias in Experiments 5a and 5b provided evidence of a strong strong wave of attention guidance, present by 400 ms asymptoting by 1,000 ms at around 50% Target Bias. Note that while this was not consistent with *continuous* guidance toward a single target, it *was* consistent with guidance (by around 700–1,000 ms) toward one of two or three candidate targets, this selected item being the target on 50% of occasions in a target-present display. Accordingly, these patterns were consistent with global or local guidance.

The finding of PARAglobal processing (or SERIALglobal with attention to large clumps of greater than three items) – i.e., fairly stable broad attention and global guidance – was consistent with evidence that in visual search displays, attention guidance tends to be associated with broad attention (prior to identification of a candidate target). Attention guidance by singletons outside the focus of spatial attention, appears to be limited, even by salient, ‘pop-out’ singletons (Burnham, 2018; Ruthruff & Gaspelin, 2018; Theeuwes et al., 2001; see also Berggren & Eimer, 2020, for event-related potential evidence and recent review). However, note that the current findings cannot speak to the necessity of broad attention for guidance.

## General discussion

This work trialled an alternative to set size in visual search: the Attention Location and Size (ATLAS) Task. To assay characteristics of covert attention, performance of search tasks was interrupted unpredictably by a Memory Display, then replaced by a Choice Display, in which participants chose (from a limited ‘Choice Set’) the location of the Memory Display item they remembered best. ATLAS, in contrast to standard set-size measures, kept Search Display characteristics constant across conditions, varying, instead, participants’ choices over which memory items they reported. Location of peak attention and attention breadth were indexed during search in eight initial experiments. Patterns of 1ofN, 1of1 and NofN scores distinguished reasonably well between studies expected to require broad, parallel versus narrow, serial attention (though future work should develop more sensitive measures) and a ‘Pairwise Attention Indicator’ appeared to detect selection of pairs of items during search, within the particular search stimuli examined here.

In Experiments 5a and 5b, designed to elicit strong top-down guidance, the strength and distribution of guidance was indexed across SOAs and qualitatively compared to estimates of spatial attention properties. From 400 ms onwards, steady, *global* guidance of attention was evident, accompanied by evidence of broad attention. The current results suggest that ATLAS will be useful for characterising properties and guidance of spatial attention during search, helping resolve thorny issues regarding whether a given manipulation in visual search improves search efficiency by enhancing speed of serial attention or improving guidance. Of course, this approach is limited, too. Not all processes in search need be expressed in the control of attention and other approaches, such as those of Townsend and colleagues, may offer better access to those processes. Additionally, it may yet be that limited temporal precision of attention probes could permit very fast serial processes to mimic slower, clump-based attention processes. However, for the types of practical question often facing authors of visual search studies, ATLAS offers one means of addressing questions of interest.

This initial paper on ATLAS did also not address in detail attempts to resolve serial-parallel questions on the basis of search slopes (e.g., Sung, 2008) as they are based on set size manipulations. Similarly, there was no discussion physiological measures of attention (e.g., Li et al., 2020), or the role of ‘items’ versus fixations (Hulleman & Olivers, 2017; Zelinsky & Sheinberg, 1997); this was simply to limit the scope of the article. Other behavioural measures addressing serial-parallel processing distinctions were addressed, and those may be combined with the approach espoused here, within individual experiments. Notably, both the approach of Townsend and colleagues (e.g., Townsend et al., 2011) and Lee and colleagues (Lee et al., 2021) require the search task to comprise two targets. This and other potential limitations discussed earlier, motivated the alternative approach piloted here: as the limitations of ATLAS are different, the two approaches may be mutually supportive where there is agreement on a particular interpretation.

Another common method, sometimes applied to visual search, uses variations on Shiffrin and Gardner’s (1972) simultaneous-sequential paradigm. In each trial, all display items are either presented at one (‘Simultaneous Condition’), or only half the items are presented, then the remaining ones in a subsequent display (‘Sequential Condition’ – e.g., Attarha et al., 2014). This approach elegantly equates some aspects of processing across set sizes (in particular, overall memory load) in briefly presented displays. However, the two conditions are in other respects quite dissimilar. In sequential condition displays, only half the number of items onset at any one time relative to the simultaneous condition (retaining an essential limitation of set size). Moreover, spatial attention is also forcibly spread across a larger region in the simultaneous condition. This latter limitation indeed applies to any manipulation that effectively varies set size by cueing attention to a subset of the display, or by making some of the nontargets easy to reject and varying the proportion of those.

A further limitation of this paradigm, explicitly acknowledged here, is that it (like other behavioural paradigms intended to measure spatial attention during search) is dual-task, requiring maintenance of two task sets and switching between them, plus potential interactions between perception and memory loads related to each task. To measure spatial attention elicited by a Search- or a Spatial-Monitoring task using the Memory Task, while controlling approximately for such variables, most analyses here compared accuracy in one condition relative to another, rather than absolute accuracy. Nonetheless, it must be conceded that these attempts to control for dual-task costs may not have succeeded and certainly cannot remove the dual-task components from the paradigm, which remain limitations. These shortcomings might eventually be addressed using M/EEG to provide passive indices of covert attention (see, e.g., Luck et al., 1993). However, whether such measures could provide a clear index of attention breadth *when the centre of the attention window is unknown*, remains unclear. I have not discussed these possibilities here as the current work focussed solely on behavioural indices.

### Stimulus specificity of the current findings

As noted in the Introduction, the analyses here concerned only *one type* of search stimulus array – displays of six small kite shapes that were regularly spaced. While it was important, here, to establish in one example set of search stimuli that ATLAS could be used to distinguish broad from narrow attention, and broad attention guidance, the use of a single type of search array means that the conclusions here are only evidenced with respect to those stimuli. It remains unclear whether the same patterns will be observed in other types of stimuli, and additionally whether changes in set size, item size and complexity or in the regularity and size of inter-item distances, will alter the patterns of findings observed here. If performance signatures associated with parallel and serial attention are different for different search stimuli, this might impose limits on the usefulness of ATLAS exactly as described here.

To address this lack of generality, future work should seek to generalise ATLAS to a range of visual search stimuli. This may be a lengthy process as we should not assume that the same patterns of Memory Task performance will arise for each stimulus set. Rather, *for each new set of search stimuli*, a clear expected set of patterns of Memory Task performance should be established for serial and parallel attention against which to compare performance in the search task of interest. This can be established empirically as the current work has attempted to do, by again conducting experiments with those stimuli where either parallel or serial attention might be (intuitively) assumed. Providing such other patterns of results make intuitive sense, this need not limit this approach’s generalisability, even if they differ from the current pattern.

### A continued role for set size

As discussed in the *Introduction*, the visual search task is not only used to investigate the role of attention. Target detection amongst of stimuli of varying complexity, heterogeneity and other factors are of widespread interest (e.g., Buetti et al., 2016; Wolfe & Horowitz, 2017) irrespective of the particular roles of attention and guidance processes. For such work, on effects of *perceptual* variables in the display (e.g., crowding: Veríssimo et al., 2021) or incidental learning of display patterns (e.g., Zhang & Carlisle, 2023), much simpler (to run) standard set size manipulations should probably still be preferred. The downside of attempting to measure the properties of *covert* attention and its guidance (as ATLAS and other approaches have shown) is that these seem to require more complex sets of conditions than set size. ATLAS is a much more complex and unwieldy relative to set size: hence, when modes of attention are not of key interest, ATLAS in its current form cannot be recommended over set size for studying effects of perceptual variables on search efficiency, with its restrictions on placement and potential shapes of Search Display items. This should not, however, deter future research from examining whether ATLAS might be adapted to suit those less regular types of stimuli.

### Potential effects of eye movements

As briefly discussed in the General Methods, the Memory Task items used here were explicitly designed to minimise the need for fine acuity and hence any effects of where the participant happened to be fixating when the Memory Display was presented. One result that suggests ATLAS measures were indeed robust to effects of eye movements is that results for 150 ms SOA in Experiment 5b were very similar to those at longer SOAs (we have found the same pattern in another unpublished study designed to guarantee serial search). If results before most saccades begin are indistinguishable from those at longer SOAs, this suggests a relatively minor role for fixation position.

Nonetheless, future work should test ATLAS either by instructing participants to maintain fixation (using eye-tracking to exclude trials with large saccades), by minimising the potential for eye-movements (e.g., Smith et al., 2014) or by examining the effects of current fixation position on performance. The former of these approaches necessarily reduces ecological-validity of the search processes observed and cannot perfectly control for saccade/fixation-position effects as these will, to some degree, correlate with the position of covert attention (e.g., Williams & Pollatsek, 2007). Nonetheless, if these effects prove relatively minor (as suggested by stable patterns across SOAs, described above), it should be possible to demonstrate this more fully in future work.

### Why did NofN-1ofN scores not distinguish broad from serial attention?

One unexpected result here was that NofN-1ofN scores, expected to be smaller for parallel than for serial attention, did not differ noticeably. One possibility is that this finding reflected strong exogenous cueing, by Memory Display elements, that overwhelmed inter-experiment differences in attention breadth. However, that explanation is not consistent with the apparent efficacy of 3ofN:Alt versus 3ofN:Adj measures in detecting narrower attention to two items (where cueing effects would be expected to homogenise performance on the same basis). Similarly, it does not seem likely that retro-cueing properties of the Choice Display for 1ofN trials obscured NofN-1ofN score differences for Serial versus Parallel Experiments; they should also have done so for 1of1 trials and affected those comparisons.

An alternative possibility is that the Serial tasks employed here might have limited NofN performance particularly. For example, in Experiment 1, a single, attended item at any point during search might have appeared at one of four orientations, so might typically have required mental rotation of the item to determine if it is the target. The ‘Serial Pairs’ tasks also potentially required mental transformation processes to be applied to each pair of items to compare them. These processes might have adversely affected perception of a Memory Task item appearing at the attended location, but not other locations. That would have adversely affected NofN performance relative to other conditions (as NofN always involves reporting of the item at the attended location). This could therefore account for two puzzling features here. First, it could explain why NofN-1ofN scores are smaller in the Serial tasks than might be expected (due to reduced NofN scores), relative to Parallel tasks. Second, it would explain why NofN accuracy in the Parallel tasks was higher than in the Serial tasks.

A third potential explanation is that while the 1of1 condition was sensitive to thinly spread attention resource (notionally present in Parallel, but not Serial Experiments), the 1ofN condition was not, due to effects of inter-item competition among Memory Display items in the 1ofN Condition. Perhaps, contrary to the author’s intention when designing the display, when attention was notionally ‘broadly spread’ in the Parallel experiments, participants’ spatial attention distribution may have collapsed when six-item Memory Displays were presented, randomly falling onto just one or two of the previously attended items. This would, without threatening the validity of the other current measures, account for the approximate equivalence of NofN-1ofN scores for parallel versus serial experiments here.

These two latter accounts are not mutually exclusive. Future work should distinguish which of these possible accounts (or another account) best explains this unexpected result, so that NofN-1ofN scores can also be clearly related to elements of attention breadth. While this may mean a shifting focus from NofN-1of1 scores to include 1ofN accuracy, this should not threaten the validity of the current approach.

## Concluding remarks

In an example set of search stimuli here, ATLAS measures appeared to distinguish broad- from narrow attention, identified pairwise attention (at least in one experiment) and indexed rapid guidance of attention during search. These findings motivate further work to examine the generalisability of ATLAS to distinguish the five modes of search described. Little critical analysis of other measures of serial versus parallel search has been offered in this initial investigation, partly to limit the already substantial length of the article, but partly because there is no need for exclusivity of tools to contribute to this topic. Each can be seen as toolbox components with complementary strengths. A strength of ATLAS is that, in principle, given the caveats outlined here, it can be applied to visual searches for one target among a range of nontargets (at least, in the stimuli studied here). On the other hand, it brings its own disruption to search: performance measures rarely avoid them. ATLAS as described here also is not yet as sensitive to differences in breadth of attention as it might be and does not provide a parameter estimate of attention breadth or efficiency of guidance.

There is clearly more development work to do. ATLAS as described here might therefore be considered ‘Mk 1’, signalling as aspiration to augment its sensitivity and the precision of its estimates in future variants. These shortcomings aside, it seems better placed than set size, to profile the attention and guidance mechanisms at play in search tasks.

## Data Availability

Data and project materials are available via the Open Science Framework at https://osf.io/s6xdc/?view_only=a71848ca0cf14cd694f676bd661de8b3

## Code availability

Experiments are available at: https://app.gorilla.sc/openmaterials/733395
